# Vitamin D, and Maternal and Child Health

**DOI:** 10.1007/s00223-019-00560-x

**Published:** 2019-05-14

**Authors:** Rebecca J. Moon, Justin H. Davies, Cyrus Cooper, Nicholas C. Harvey

**Affiliations:** 1 MRC Lifecourse Epidemiology Unit, Southampton General Hospital, University of Southampton, Southampton, SO16 6YD UK; 2 Paediatric Endocrinology, University Hospitals Southampton NHS Foundation Trust, Southampton, UK; 3 National Institute for Health Research (NIHR) Southampton Nutrition Biomedical Research Centre, University of Southampton and University Hospital Southampton NHS Foundation Trust, Southampton, UK; 4 National Institute for Health Research (NIHR) Biomedical Research Centre, University of Oxford, Oxford, UK

**Keywords:** Vitamin D, Rickets, Pregnancy, Bone mineral density, Supplementation

## Abstract

Vitamin D has important roles in calcium metabolism and in the prevention of rickets and osteomalacia; low levels of 25-hydroxyvitamin D are common in the general population and amongst pregnant women. Whilst there is a wealth of observational evidence linking vitamin D deficiency to a wide range of disease outcomes, there are currently few high-quality randomised controlled trials to confirm any causal associations, although many are currently in progress. Furthermore, currently, the vast majority of published guidelines recommend standard supplemental vitamin D doses for children and pregnant women, yet there is increasing recognition that individual characteristics and genetic factors may influence the response to supplementation. As such, future research needs to concentrate on documenting definite beneficial clinical outcomes of vitamin D supplementation, and establishing personalised dosing schedules and demonstrating effective approaches to optimising initiation and adherence.

## Introduction

Vitamin D, along with calcium, is important for skeletal growth and bone health, and may also influence soft tissue body composition, foetal development and obstetric health. This article will review the evidence to support routine vitamin D supplementation in childhood and pregnancy.

## Vitamin D

Vitamin D is a secosteroid, which can be obtained from the diet as either ergocalciferol (vitamin D_2_) from plant sources or cholecalciferol (vitamin D_3_) from animal sources. Vitamin D can also be synthesised endogenously within the skin when 7-dehydrocholesterol is exposed to ultraviolet B (UVB) irradiation. Vitamin D is converted to 25-hydroxyvitamin D [25(OH)D] by 25-hydroxylase in the liver. It is the main circulating form of vitamin D and acts as a reservoir for conversion to the active metabolite 1,25-dihydroxyvitamin D [1,25(OH)_2_D] by 1α-hydroxylase. This enzyme is primarily located in the renal proximal tubular cells, but also to a lesser extent in the placenta, bone and parathyroid gland.

The classical function of vitamin D is in calcium and phosphate homeostasis. Synthesis of 1,25(OH)_2_D within the kidney is tightly regulated in response to serum ionised calcium (Ca^2+^) levels. This occurs in conjunction with parathyroid hormone (PTH) and fibroblast growth factor-23 (FGF-23). Low Ca^2+^ stimulates the release of PTH, which simultaneously increases renal calcium reabsorption in the distal tubule of the kidney, decreases proximal tubule phosphate reabsorption and upregulates 1,25(OH)_2_D synthesis. The main action of 1,25(OH)_2_D is to increase the uptake of dietary calcium through the intestinal enterocytes. PTH also promotes bone resorption by osteoclasts, thereby mobilising calcium and phosphate from bone mineral. However, the presence of 1,25(OH)_2_D is required for this to occur. 1,25(OH)_2_D also increases the production of FGF-23, which acts to increase urinary phosphate excretion and downregulate 1α-hydroxylase activity and PTH release. As such, low levels of vitamin D can result in poor intestinal calcium absorption, and subsequently a reduction in serum Ca^2+^. This leads to secondary hyperparathyroidism, with subsequent mobilisation of bone mineral, increased renal calcium reabsorption, but also increased urinary phosphate wasting. In early vitamin D deficiency (VDD), serum calcium concentration is usually maintained within the normal range, but phosphate is often low. As skeletal calcium stores are depleted, hypocalcaemia may ensue.

The actions of vitamin D are mediated through the vitamin D receptor (VDR), and occur through both slow genomic actions, for example, transcription of intestinal calcium channels, and more rapid non-genomic effects, including rapid intestinal calcium uptake [1]. The VDR has been isolated in a wide variety of cell types including osteoblasts, keratinocytes, macrophages, pancreatic β cells, adipose tissue and skeletal muscle [2, 3], supporting the hypothesis that vitamin D might have diverse actions apart from in calcium metabolism.

## Epidemiology of Vitamin D Deficiency

25(OH)D is currently considered the best biochemical marker of vitamin D status as hepatic hydroxylation of cholecalciferol to 25(OH)D is dependent only on substrate availability, whereas the conversion of 25(OH)D to 1,25(OH)_2_D is tightly regulated in response to serum Ca^2+^ and PTH. 25(OH)D has a significantly longer half-life than 1,25(OH)_2_D: approximately 2–3 weeks compared with 4–6 h [4].

The serum concentration of 25(OH)D that is felt to constitute adequacy is a subject of much debate and the recommended threshold used to define VDD is highly variable between guidelines and consensus statements (Table 1), and this can limit the comparison of reported prevalence between studies. The great variability in these definitions partly reflects that there does not appear to be a single threshold below which secondary hyperparathyroidism or clinical outcomes, such as metabolic bone disease, always occur in either adults [5, 6] or children [7]. This is likely due to the interactions between vitamin D and dietary calcium and magnesium intake, ethnicity and vitamin D binding protein (DBP) genotype, which will modify the association between 25(OH)D and PTH [8, 9]. Perhaps most importantly, recognition that VDD is not synonymous with rickets in children or osteomalacia in adults is needed, particularly in the lay media, and indeed in some individuals a biochemically low serum 25(OH)D might not be associated with detrimental clinical outcomes.

**Table 1:** Definitions of vitamin D deficiency, insufficiency and sufficiency according to a number of recent guidelines and consensus statements

Guidelines	Deficiency (nmol/l)	Insufficiency (nmol/l)	Sufficiency (nmol/l)
Institute of Medicine, IOM [134]	< 30	30–50	≥ 50
Endocrine Society Practice Guidelines [135]	< 50	50–75	≥ 75
Scientific Advisory Committee on Nutrition (SACN) and UK Department for Health [136]	< 25		≥ 25
British Paediatric and Adolescent Bone Group [151]	< 25	25–50	≥ 50
Global Consensus Recommendations on Prevention and Management of Nutritional Rickets [137]	< 30	30–50	≥ 50
National Osteoporosis Society (UK) [152]	< 25	25–50	> 50
Canadian Paediatric Society [138]	< 25	25–75	75–225
Working Group of the Australian and New Zealand Bone and Mineral Society, Endocrine Society of Australia and Osteoporosis Australia [153]	< 50		≥ 50 At the end of winter (level may need to be 10–20 nmol/l higher at the end of summer)

**Fig. 1:**
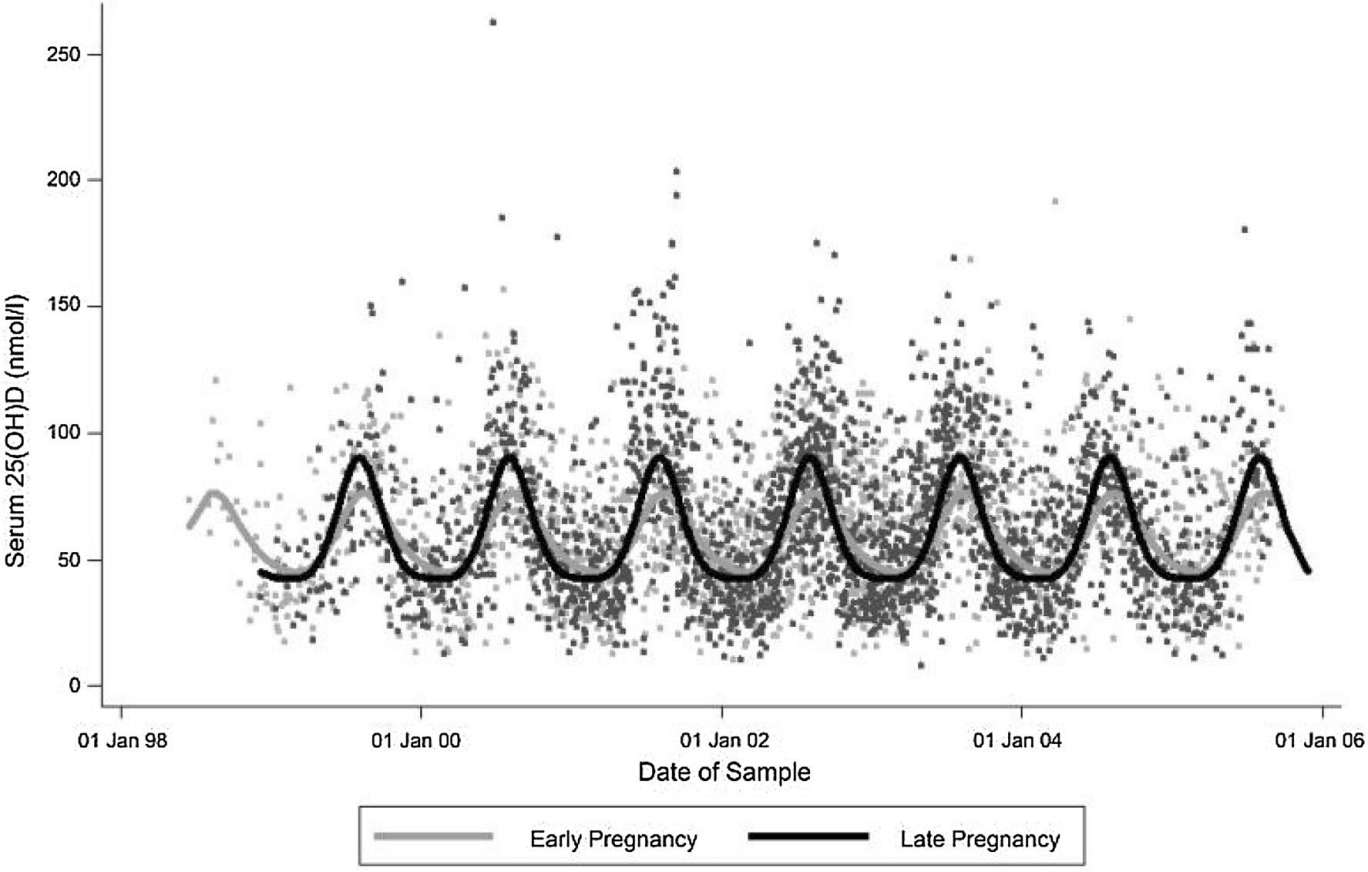
Seasonal variation in maternal serum 25-hydroxyvitamin D status in early and late pregnancy in an observational cohort of women in Southampton, UK (50.9°N). From Moon et al. [15]

There are few data which document the epidemiology of serum 25(OH)D concentrations across the general population, but studies in selected groups often report a high prevalence of VDD. Two recent large studies in the United Kingdom (UK) demonstrated that around a third of children had a serum 25(OH)D < 50 nmol/l [10, 11], and a similar prevalence has also been reported in pregnant women in the UK [12–14]. A number of risk factors for VDD have been consistently identified, many of which are related to reduced UVB exposure and limited cutaneous synthesis of cholecalciferol. In high latitude countries, there is marked seasonal variation in serum 25(OH)D status, being lowest in late winter and peaking in mid-late summer months (Fig. 1) [10, 11, 15]. Furthermore, the effect of latitude on 25(OH)D status is even observed within the UK with children and post-menopausal women residing in Northern England and Scotland having lower 25(OH)D levels than those living in the south of England [10, 16]. As such, at northern latitudes, individuals are more reliant on dietary sources and supplementation to prevent VDD during winter months [10, 17]. Furthermore, even at the same latitude, serum 25(OH)D levels are consistently higher in white compared with dark-skinned individuals [10, 16–18]. Greater outdoor time and play is protective against VDD [10, 11], whereas extensive skin covering for religious or cultural reasons and liberal use of sun protection prevent cutaneous vitamin D synthesis [19]. Greater social deprivation, including lower parental educational achievement and income and living in rented housing have also been associated with lower 25(OH)D status in childhood [10, 11, 17, 18]. The mechanisms for this may include lower outdoor leisure time, sunlight exposure or poorer dietary intake and supplementation use [15].

## Vitamin D Deficiency, Rickets and Childhood Bone Health

Rickets and osteomalacia are the bony consequences of severe VDD. Rickets is a disorder of growth plate ossification and mineralisation, which occurs only in growing bone. VDD is one of the number of biochemical and/or hormonal disturbances (e.g. calcium deficiency, phosphate deficiency) which results in the clinical phenotype of rickets. Hypophosphatemia is the common end pathway in these biochemical abnormalities, and is postulated to be the cause of the rachitic changes in the growth plate by reducing apoptosis of the hypertrophic chondrocytes and leading to poor mineralisation of the metaphysis [20]. Following fusion of the growth plates, VDD can result in osteomalacia, in which there is undermineralisation of the osteoid. This is a histological diagnosis that can only definitively be made by bone biopsy, although treatment with vitamin D replacement is usually commenced when clinical features including bone pain, muscle weakness and pathological fractures are present with a low serum 25(OH)D.

The clinical features of rickets are variable and in part dependent on the developmental age of the child. The classical skeletal features include bony swelling at the wrists, knees, ankles and costochondral junction (rachitic rosary), and in mobile children bending of the limbs might be observed. Linear growth, dentition and motor development are also commonly delayed. In the neonatal period and in adolescence, when calcium demands are higher to meet the demands for faster linear growth, hypocalcaemic seizures can be the first presenting feature of rickets [21]. Dilated cardiomyopathy has also been reported [22].

In many developing countries, there is a suggestion of a rising incidence of rickets [23–27]. Recent surveys in the UK, Australia, Japan and Canada have reported the incidence of symptomatic VDD (radiographic rickets or hypocalcaemic seizures due to VDD) to be 1.1–7.5 per 100,000, although the incidence varies widely between ethnic groups [28–32]. A 2001 survey of VDD rickets in children aged less than 5 years in the West Midlands, UK, estimated the incidence in White children to be 0.4 per 100,000 compared with 38 per 100,000 in Asian children and 95 per 100,000 in children of Black-African or Afro-Caribbean ethnicity [28]. The overall increasing incidence of rickets in many countries might therefore in part reflect demographic changes, as demonstrated in a study conducted in southern Denmark where the overall incidence of VDD rickets had increased in 1995–2005 compared with 1985–1994, yet the incidence actually decreased in ethnic Danish children [33]. As such, VDD rickets is not a rare disease, but how frequently a clinician encounters it in clinical practice will be highly dependent on the demographics of the population served.

Although there is a clear causal role for VDD in nutritional rickets, which suggests the need for routine supplementation, the importance of biochemically low 25(OH)D levels in subclinical bone health outcomes including low bone mineral density (BMD) and fractures remains to be established [34]. Early infancy has been a period of particular interest due to the low vitamin D content in maternal breast milk, yet intervention studies of vitamin D supplementation in breast fed infants have not identified significant effects on measures of bone mineral content (BMC), BMD or bone strength [35–37]. In childhood and adolescence, cross-sectional studies examining the associations between 25(OH)D status and BMD have reported inconsistent findings when 25(OH)D is considered as a continuous variable, but the use of 25(OH)D as a dichotomised variable has typically resulted in those with the lowest levels of serum 25(OH)D having significantly lower BMD at one or more skeletal sites [34]. Given that vitamin D is primarily obtained from sunlight exposure, the interpretation of observational studies relating vitamin D to any outcome is limited by reverse causality and confounding, particularly from outdoor physical activity. Indeed, objectively assessed physical activity using accelerometry is positively associated with serum 25(OH)D status in children and adolescents [38], but physical activity also has beneficial effects on bone mineralisation. Interestingly, the exception to the findings of studies relating 25(OH)D to BMD in adolescence were those based on cohorts of ballet dancers, in whom 25(OH)D was negatively associated with BMD, but confounding by high levels of indoor physical activity is likely [39, 40]. A non-linear effect of vitamin D supplementation on BMD has also been suggested by a meta-analysis of 6 randomised controlled trials (RCTs) conducted in children and adolescents. Winzenberg et al. found that vitamin D supplementation did not lead to significant gains in whole-body BMC, forearm, hip or lumbar spine BMD overall, but in studies in which baseline serum 25(OH)D was less than 35 nmol/l, supplementation resulted in 2.6% and 1.7% greater change in whole-body BMC and lumbar spine BMD, respectively, compared to placebo [41]. This was not observed in studies with higher baseline serum 25(OH)D, but further replication in larger studies is required, and the authors of this meta-analysis have proposed an individual patient data meta-analysis to further explore the relationships [42].

Fracture is common in childhood [43], and public health approaches to reduce the burden of childhood fractures are needed, yet there is little evidence to support a role for vitamin D supplementation in this at the current time. The few published case–control observational studies have not found a significant difference in serum 25(OH)D in children with a fracture and controls [34, 44], although Anderson et al. reported self-selected vitamin D supplementation use reduced the risk of fracture in children aged < 6 years by 58% [44]. Adjustment for skin type, waist circumference, outdoor free play time, parental income, birth weight and soda intake was made, but it is possible that this finding reflects residual confounding by other lifestyle, dietary or parenting factors. To date there are no intervention studies of vitamin D supplementation to reduce fracture but the size of the study needed and duration of follow-up limit the likelihood of an RCT being undertaken.

## Vitamin D, Obesity and Muscle Function in Childhood

There is a wealth of data that consistently demonstrates negative associations between serum 25(OH)D concentration and various measures of adiposity, including body mass index (BMI) and fat mass (FM) [45, 46]. Interestingly, PTH is positively correlated with BMI and total FM [47], suggesting a functional effect of VDD in obesity that warrants treatment with vitamin D supplementation; chronic excess PTH can lead to insulin resistance and has been hypothesised to promote adipogenesis [48]. But despite this, the available evidence to date suggests that obesity is a cause rather than consequence of VDD. Firstly, a double-blind RCT in childhood and adolescence demonstrated no significant difference in BMI, BMI *z*-score or waist circumference following 3 or 6 months of 4000 IU/day cholecalciferol compared to placebo despite a significant increase in 25(OH)D [49]; intervention studies in adult populations have also not identified significant effects of vitamin D supplementation on BMI or FM [50]. Secondly, serum 25(OH)D increased to a lesser extent in obese compared to non-obese individuals following total body UVB irradiation despite a similar capacity of the skin to synthesise vitamin D, suggesting sequestration outside of the circulating pool, likely in adipose tissue due to the lipophilic nature of 25(OH)D [51]. A lower increase in 25(OH)D is also observed in response to the same dose of vitamin D in obese compared with non-obese individuals [52, 53] and in 67 participants of an adolescent obesity weight loss intervention programme, a significant relationship was identified between change in BMI *z*-score and change in 25(OH)D status [54]. Mendelian randomisation has also been used to demonstrate that single-nucleotide polymorphisms (SNPs) associated with higher BMI are also associated with lower 25(OH)D, whereas SNPs known to determine 25(OH)D status were not associated with BMI, further suggesting that VDD occurs secondary to obesity [55]. Overall, there is little evidence to support the use of vitamin D supplementation to prevent or treat obesity, but awareness that this population group is more likely to be vitamin D deficient and requires supplementation to achieve recommended 25(OH)D levels is important.

Myopathy, myalgia and developmental delay can be presenting features of VDD and often improve following vitamin D supplementation [23, 30, 56], but whether these symptoms occur as a direct result of low serum 25(OH)D or due to other biochemical disturbances secondary to this, e.g. hypophosphatemia or hypocalcemia, is not currently understood. In elderly subjects, histological studies have demonstrated atrophy of type II muscle fibres in VDD [57] and an increase in type II fibre size and number following supplementation with ergocalciferol [58], but similar studies have not been undertaken in young subjects. Lean mass and muscle strength are important outcomes, even for healthy children. Muscle mass increases throughout childhood to peak at approximately 18 to 20 years of age, followed by a subsequent decline due to myofibre loss. Grip strength follows a similar pattern [59]. Both low muscle mass and lower grip strength in adulthood have been prospectively associated with worse clinical outcomes including all-cause mortality, non-cardiovascular mortality and myocardial infarction [60], and in childhood and adolescence, grip strength has been negatively associated with blood pressure and positively associated with insulin sensitivity after adjustment for cardiorespiratory fitness [61]. As such, interventions to increase muscle size and strength could be beneficial to overall public health. Observational studies have demonstrated positive associations between 25(OH)D status and measures of muscle mass [62], muscle strength and power in adolescents [63, 64], but similar to the observations between 25(OH)D and bone mass, reverse causality and/or confounding might underlie the relationships. To our knowledge, there is only one trial of vitamin D supplementation in adolescence examining the effect on muscle function: Ward et al. randomised 72 ethnically diverse post-menarchal girls aged 12–14 years to 150,000 IU oral ergocalciferol or placebo every 3 months for 1 year. At the end of the study, jumping height and velocity and hand-grip strength were numerically greater in the girls randomised to vitamin D but these did not reach statistical significance [65]. Intervention studies in older adults have demonstrated small but statistically significant beneficial effects of vitamin D supplementation on muscle strength [66], highlighting the need for further data in younger subjects.

## Vitamin D and Pregnancy

Similar to the situation in childhood, VDD is common in pregnancy. In the Princess Anne Hospital Study, UK, which included women predominantly of white ethnicity in the UK, 31% had a serum 25(OH)D < 50 nmol/l and 18% < 25 nmol/l at 34 weeks of gestation [13], but levels considered to be deficient are even more prevalent in ethnically diverse populations [14].

Alterations to maternal calcium and phosphate metabolism occur during pregnancy to meet the demands for foetal mineral accretion. The foetal skeleton contains approximately 30 g of calcium by the end of pregnancy, the majority of which is obtained during the last trimester [67]. This occurs through both increased maternal intestinal calcium absorption [68] and mobilisation of the maternal skeleton [69], but without alteration to maternal serum Ca^2+^ concentration. Maternal calcitropic hormones are likely to have an important role in these adaptations. Total 1,25(OH)_2_D increases early in pregnancy [68, 70, 71]. DBP also increases early in pregnancy, but unlike 1,25(OH)_2_D does not continue to increase in late pregnancy, thus free 1,25(OH)_2_D is increased in the third trimester relative to earlier in pregnancy [71]. This increase in 1,25(OH)_2_D appears to be independent of PTH, which remains within the normal adult range throughout pregnancy [67]. However PTH-related protein is elevated in the maternal circulation from early pregnancy [70] and might contribute to the rise in 1,25(OH)_2_D. Despite the increase in 1,25(OH)_2_D, the effect of pregnancy on 25(OH)D status is less well understood. There are few longitudinal data and most are from small studies including between 10 and 40 women. Moreover, the findings are contradictory, and are likely influenced by timing of participant recruitment and the expected seasonal variation in 25(OH)D [68, 69, 72]. Nonetheless, it is evident that women who have low levels of 25(OH)D in early pregnancy are likely to continue to have low levels in late pregnancy also [15], highlighting that supplementation should be encouraged in women at risk of VDD.

## Maternal Vitamin D Deficiency and Offspring Health and Development

The foetus is dependent on the mother for 25(OH)D. 25(OH)D will readily cross the placenta, and maternal and umbilical cord venous blood 25(OH)D are moderate-highly correlated [73–75]. It is clear that antenatal vitamin D supplementation can increase umbilical cord venous and neonatal serum 25(OH)D compared to placebo [74, 76–79], and that larger oral doses of cholecalciferol (1000–4000 IU/day) result in higher umbilical cord venous or neonatal serum 25(OH)D when compared to supplementation with 400 IU/day [80, 81].

Maternal VDD can result in neonatal hypocalcaemia. Clinically, this can result in seizures, and has been associated with craniotabes [82], and rarely dilated cardiomyopathy [83]. There is consistent evidence that vitamin D supplementation in pregnancy can reduce the incidence of symptomatic hypocalcaemia in the neonate [76, 84, 85], and this alone may justify the use of routine supplementation in pregnancy. There is also increasing evidence to suggest that vitamin D might have a more diverse role in programming offspring development and reduce maternal obstetric complications, and as a result there has been an exponential increase in interventional trials of antenatal vitamin D supplementation in recent years [86].

### Birth Anthropometry and Childhood Growth

Size at birth is one of the most commonly reported outcomes in both observational studies of maternal 25(OH)D status in pregnancy and interventional studies of antenatal vitamin D supplementation [86, 87]. There is considerable heterogeneity in observational studies, including study size, maternal gestation at assessment and population demographics, but when considered together, these studies would suggest that foetal size is only affected at the lowest levels of maternal 25(OH)D and the relationship is non-linear. Indeed, studies which have considered 25(OH)D as a continuous variable have not typically identified a significant relationship between maternal 25(OH)D and offspring birth weight or length [73, 75, 88–97]. In contrast, dichotomisation of maternal serum 25(OH)D to compare two or more groups identified significantly lower birth weight in babies born to mothers who were classed as VDD when thresholds between 25 and 37.5 nmol/l were used to define VDD, but not in studies that used a higher serum 25(OH)D to define VDD [75, 89, 90, 98–103]. The largest of these studies included historical data from 2146 participants in the Collaborative Perinatal Project (CPP) in the USA and assessed 25(OH)D before 26 weeks of pregnancy. When maternal 25(OH)D was less than 37.5 nmol/l, 25(OH)D was positively associated with offspring birth weight (*β* = 3.6 g/nmol/l [95% CI 1.1, 6.1]) and occipitofrontal circumference (0.01 cm/nmol/l [95% CI 0.002, 0.018]), but there was no significant association between maternal 25(OH)D and offspring size for 25(OH)D concentrations above 37.5 nmol/l [100].

Meta-analysis of thirty trials of antenatal vitamin D supplementation published before 2017 identified that antenatal vitamin D supplementation (compared to low dose, no vitamin D or placebo) increased mean birth weight by an average of 58 g [86]. Sensitivity analysis found an effect on birth weight when bolus dosing of vitamin D was used but not with daily dosing, which could reflect improved compliance, or achievement of higher 25(OH)D levels, yet no dose–response effect was identified, and an effect in women with lower baseline 25(OH)D was not observed [86]. These findings are therefore not consistent with the suggestion from observational studies of supplementation being particularly important to women who have biochemically low levels of 25(OH)D.

To date, intervention studies also suggest a positive effect of maternal vitamin D supplementation during pregnancy on postnatal growth. The earliest study was conducted by Brooke et al. in 1981 and found that despite no differences in birth weight or length, infants of Asian mothers living in the UK who received 1000 IU/day ergocalciferol during the last trimester of pregnancy were significantly heavier at 3, 6, 9 and 12 months of age, and had longer crown-heel length at 9 and 12 months of age than infants born to the mothers in the control group (placebo was not given) [104], although care in the interpretation of these findings is needed as maternal characteristics are not presented and therefore it is not certain that these were similar between the two groups. More recently in the Antenatal Vitamin D in Dhaka (AViDD) trial in Bangladesh, maternal supplementation with 35,000 IU/week oral cholecalciferol from 26 to 30 weeks of gestation until delivery was associated with accelerated linear growth in the first 4 weeks of postnatal life compared to placebo despite no significant difference in birth length [105]. The difference in length *z*-score persisted until 1 year of age, but without further increase in the mean difference between the two groups. This translated to a difference in sex-adjusted length of 1.1 cm (95% CI 0.06, 2.04) between the two groups at 1 year of age. No difference in weight *z*-score was observed at either age. Assessment of infant serum 25(OH)D at 2 and 4 months of age found that the higher 25(OH)D in the supplementation group observed at birth persisted at 2 months of age, but not at 4 months of age. It is therefore possible that this contributed to the accelerated postnatal growth, although interestingly previous trials of vitamin D supplementation in infancy have not demonstrated positive effects on linear growth [36, 106]. Currently, there are no published findings relating to postnatal growth in trials of antenatal vitamin D supplementation in non-Asian ethnic groups.

### Foetal Skeletal Development

Given the importance of vitamin D to bone development in postnatal life, it is perhaps unsurprising that there has been much interest in the role of vitamin D in in utero bone development. Indeed the suggestion that gestational vitamin D supplementation increases birthweight and postnatal growth would support the notion that in utero vitamin D exposure does affect skeletal development through at the very least an increase in the size of the skeletal envelope. There are several case reports of infants born to mothers with VDD, who displayed clinical signs of rickets including bony abnormalities in addition to low serum 25(OH)D from day 1 of life [107]. These rare cases represent the most extreme descriptions of in utero VDD, but there is increasing evidence that subclinical maternal vitamin D insufficiency in pregnancy might also influence offspring bone mineral accrual. The majority is, as with other outcomes, observational in nature, although in recent years trials of antenatal vitamin D supplementation with assessment of offspring bone mineralisation have been reported.

Some of the earliest data to suggest that vitamin D exposure might influence in utero bone mineral accrual used season as a proxy marker of vitamin D status. Namgung et al. found that in 71 Korean neonates, those born in summer months had 8% higher whole-body BMC after adjustment for weight than infants born in winter. Furthermore, in that cohort, neonatal 25(OH)D measured at delivery was positively correlated with whole-body BMC [108]. In contrast, the same authors found that infants in the USA who were born in the summer had lower whole-body BMC than winter-born infants [109]. The authors proposed that these differences reflect the use of vitamin D supplementation in the two populations; the uptake of supplementation is low throughout pregnancy in Korea but standard practice after the first trimester in the USA, where differences in maternal 25(OH)D by season of birth were not observed [108]. This would suggest that early pregnancy 25(OH)D status is crucial to vitamin D mineralisation, which is in contrast to several later studies with assessment of serum 25(OH)D.

Subsequent studies have used measurements of maternal or cord blood 25(OH)D as the exposure variable. Weiler et al. studied 50 neonates born in Canada between April and August. 25(OH)D was measured in venous cord blood and used to divide the infants into two groups using a cut point of 37.5 nmol/l. The infants in the low 25(OH)D group tended to be heavier and longer, but this might have reflected the greater ethnic diversity in the group compared to the group with a high 25(OH)D level. However, whole-body and femur BMC relative to body weight were significantly lower in the 18 neonates with a cord blood 25(OH)D < 37.5 nmol/l compared with 32 infants with a 25(OH)D above this cut point [110]. Similarly, Viljakainen et al., using the mean of two maternal serum 25(OH)D measurements from early pregnancy and 2 days postpartum as the assessment of maternal vitamin D status, found neonatal tibial BMC and cross-sectional area measured by peripheral quantitative computed tomography (pQCT) were 14% and 16% higher, respectively, in infants born to mothers with 25(OH)D above the median for the cohort. Although vBMD of the tibia did not differ between the two groups, the difference in BMC and CSA did persist after adjustment for weight [88]. When a subset of these children were reassessed at 14 months of age, the difference in tibial BMC was no longer present, but tibial CSA remained significantly higher in those born to mothers with higher vitamin D status in pregnancy [111]. Conversely, in 125 Gambian mother–offspring pairs, no significant relationships were observed between maternal 25(OH)D at either 20 or 36 weeks of gestation and offspring whole-body BMC or bone area at 2, 13 or 52 weeks of age [90]. In contrast to the other studies, none of the mothers had a 25(OH)D below 50 nmol/l, which is consistent with the notion that poorer skeletal mineralisation might only occur in foetuses of mothers with the lowest 25(OH)D levels.

There is some evidence to suggest that these relationships persist into childhood, although study findings are less consistent than in the neonatal period. Positive relationships between maternal 25(OH)D measured in late pregnancy and offspring whole-body and lumbar spine bone area, BMC and BMD at 9 years of age in 198 mother–offspring pairs in the Princess Anne Hospital Study (Southampton, UK) were reported by Javaid et al. [13]. Beneficial effects of vitamin D supplementation were also suggested by this study as children born to women who consumed vitamin D containing supplements had higher whole-body BMC and bone area, but not aBMD. Although the women who took supplements were self-selected, this finding was not changed by adjustment for socioeconomic status. These findings were replicated in the Southampton Women’s Survey (SWS) in 1030 mother–offspring pairs with assessment by DXA at age 6–7 years [112]. Similarly, Zhu et al. found a positive relationship between maternal 25(OH)D status at 18 weeks gestation and bone mass in young adulthood in the Raine cohort in Western Australia. Thus, after adjustment for sex, age, height and body composition at age 20 years, maternal height and pre-pregnancy weight, maternal age at delivery, parity, education, ethnicity, smoking during pregnancy and season of maternal blood sampling, whole-body BMC and aBMD were 2.7% and 1.7% lower at 20 years of age in offspring of mothers with 25(OH)D < 50 nmol/l compared to those above this level [113]. In contrast, analyses using the Avon Longitudinal Study of Parents And Children (ALSPAC) cohort study do not support these studies. Initially, using 6995 mother–offspring pairs, Sayer et al. reported a positive relationship between estimated maternal UVB exposure in late pregnancy and offspring WBLH BMC, bone area and BMD at 9.9 years of age [114]. However, further analysis in a subset of 3960 of the children for whom maternal serum 25(OH)D measurement was available in the first (*n* = 1035), second (*n* = 879) or third (*n* = 2046) trimester did not reveal any significant associations between maternal 25(OH)D and offspring bone mineralisation [115]. Collinearity of the estimated maternal UVB measurement with age at DXA assessment is likely to have confounded the relationships reported in the initial study, and makes the findings difficult to interpret. In contrast, Garcia et al. reported an inverse relationship between maternal 25(OH)D concentration and bone mass at 6 years of age in the Generation R cohort [116], but the statistical models included both season and ambient sunshine in the month prior to blood sampling, which as the primary determinants of the exposure and not expected to be related to the outcome, may have influenced this finding [117]. Such contradictory findings only highlight the urgent need for interventional studies.

The first intervention study to assess the effect of antenatal vitamin D supplementation on offspring bone mineralisation was undertaken by Congdon et al. and published in 1983. Sixty-four women of Asian ethnicity living in the UK participated in a non-randomised study; 19 received a daily supplement containing 1000 IU vitamin D and calcium (of unknown strength) during the last trimester, whereas 45 received no supplementation. There was no significant difference in forearm BMC of the offspring at birth assessed using single-photon absorptiometry [118], but the study size, lack of randomisation and technology used limits the interpretation of the findings.

**Fig. 2:**
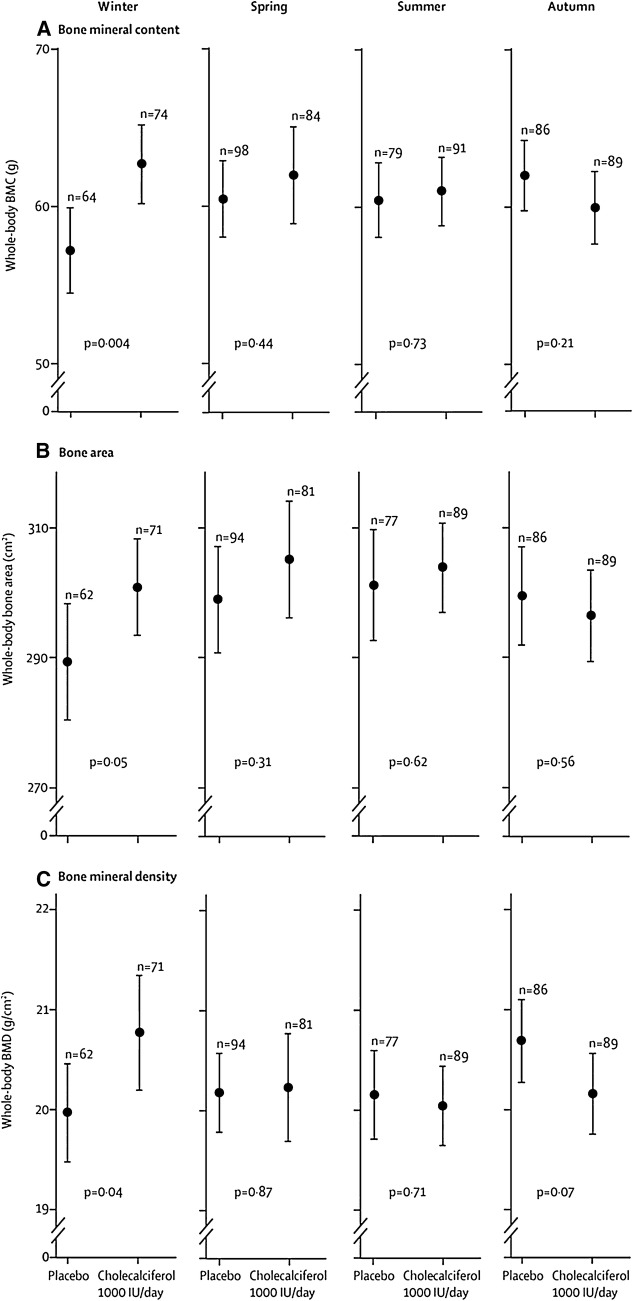
Neonatal whole-body bone mineral content (BMC), bone area and bone mineral density (BMD) by intervention group and season of birth in the MAVIDOS trial. Data are shown as mean and 95% confidence interval. Winter is December to February, spring is March to May, summer is June to August and autumn is September to November. From Cooper et al. [12]

There are three more recently published studies of gestational vitamin D supplementation, of which the largest is the Maternal Vitamin D Osteoporosis Study (MAVIDOS), a randomised double-blind placebo-controlled trial of antenatal vitamin D supplementation from 14 weeks of gestation until delivery conducted in three centres in the UK. The primary outcome was neonatal bone mass [119]. 1134 Women with a baseline 25(OH)D between 25 and 100 nmol/l were randomised to 1000 IU/day cholecalciferol or placebo; 965 remained in the study until delivery, and 736 infants had DXA of the whole body and/or lumbar spine. Although there were no differences in whole-body BMC, bone area or areal BMD between the two groups overall, a significant interaction was observed between season of birth and maternal randomisation group (*p* for interaction for BMC 0.04) [12] (Fig. 2). Thus, whole-body BMC and BMD were approximately 9% and 5% higher, respectively, in the children born in winter to mothers randomised to cholecalciferol compared to those randomised to placebo. This effect size is substantially larger than those observed between children with and without fractures [120], and hence if persisting into later childhood is likely to be clinically relevant. Follow-up of the offspring at 4 years with DXA and at 6–7 years with DXA and high-resolution pQCT is currently ongoing.

Two small intervention studies from India and Iran have also assessed bone mass in infants born to mothers randomised to vitamin D supplementation or placebo. Sahoo et al. randomised 300 women to three groups, which received 400 IU/day cholecalciferol daily (“placebo”), 60,000 IU cholecalciferol every 4 weeks or 60,000 IU cholecalciferol every 8 weeks from the second trimester. All women also received daily calcium supplementation. Only 160 women were followed up until delivery, and 52 children (17% of the original cohort) underwent DXA at 12–16 months of age. The children in the placebo group were significantly older at DXA scan and had higher measurements of whole-body BMC and BMD, but in multivariate analysis randomisation group was not a significant predictor of BMC or BMD [121]. Vaziri et al. randomised 153 women to placebo or 2000 IU/day cholecalciferol from 26 to 28 weeks until delivery, but only 25 infants (16% of the original cohort) had DXA assessment. No significant difference in whole-body BMC, BMD or bone area was found [122], but as with the study by Sahoo et al., the small numbers included are unlikely to have sufficient power to detect a difference in the outcomes studied.

Evidence from observational studies does therefore suggest that achieving higher levels of serum 25(OH)D in pregnancy might have beneficial effects on offspring bone development, but further high-quality RCTs are required to assess this. Moreover, long-term follow-up of children born to participants of these trials will determine whether any effects observed in the neonatal period, such as the effect of gestational vitamin D supplementation on increased bone mineralisation in children born in winter, does persist beyond the neonatal period.

### Offspring Soft Tissue Body Composition

**Fig. 3:**
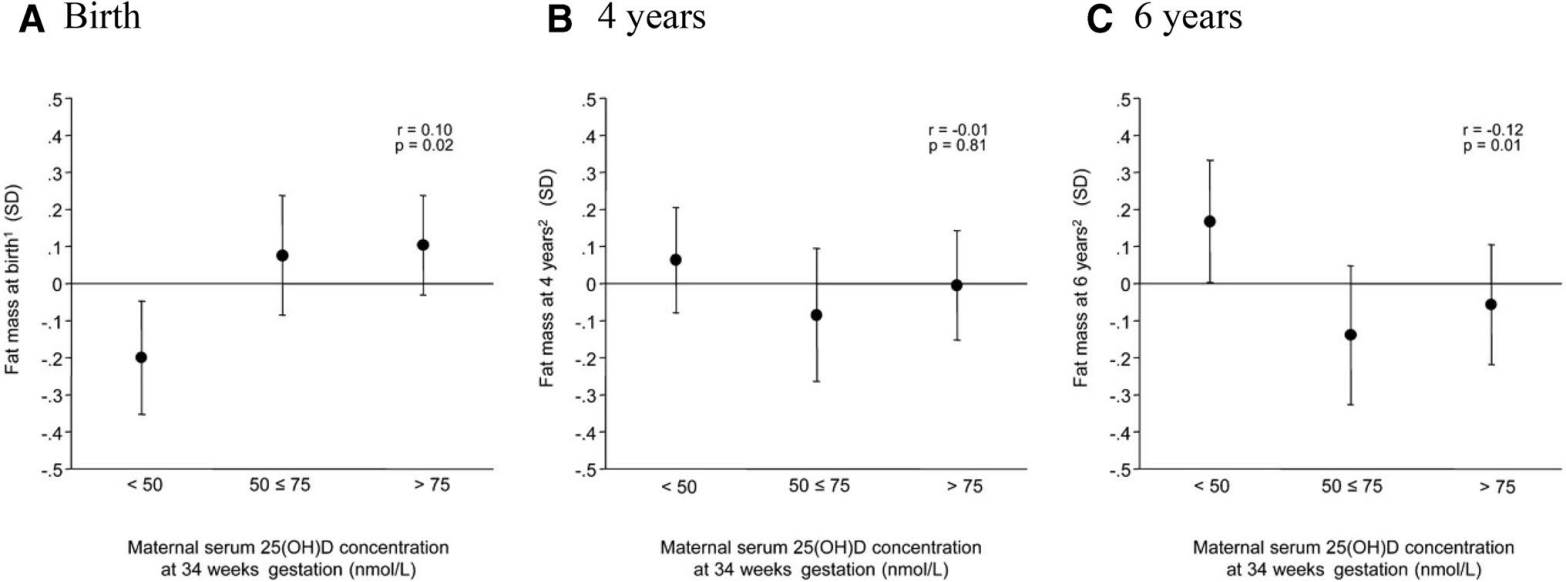
Offspring fat mass measured by dual-energy X-ray absorptiometry grouped by maternal serum 25(OH)D concentration at 34 weeks of gestation in the Southampton Women’s Survey. Displayed as mean (95% CI). From Crozier et al. [124]

Several lines of evidence suggest a possible role for vitamin D in adipogenesis and skeletal muscle development, including isolation of the VDR in human adipose tissue and skeletal muscle [123]. However, both observational data on the relationships between maternal 25(OH)D and offspring adiposity [89, 92, 103, 110, 124, 125] and the findings of two small intervention studies of vitamin D supplementation in women of Asian ethnicity that included assessment of offspring adiposity, are inconsistent [76, 126]. Interestingly, in the SWS, neonatal FM assessed by DXA was positively associated with maternal 25(OH)D in late pregnancy, but there was “flipping” of this relationship by mid-childhood. Thus, whilst no significant associations were observed at 4 years of age, at 6 years of age, maternal 25(OH)D status in late pregnancy was negatively associated with FM (Fig. 3) [124]. This finding highlights the need for further intervention studies with long-term childhood follow-up, as will be provided by the MAVIDOS study.

There are far fewer data relating maternal vitamin D status to offspring lean mass or muscle function. Children born to vitamin D replete [25(OH)D > 50 nmol/l] mothers participating in the Mysore Parthenon Study (MPS) in India, had greater arm muscle area (determined from a measurement of mid-upper arm circumference and triceps skinfold thickness) at 5 and 9.5 years than those born to vitamin D-deficient mothers [25(OH)D < 50 nmol/l], but no difference in grip strength at 9 years of age was found [127]. In contrast, in the SWS, no significant association between maternal 25(OH)D in late pregnancy and offspring whole-body or appendicular lean mass was found but a positive association with grip strength was identified [128], suggesting an effect on muscle function independent of muscle size. A 0.25 standard deviation difference in grip strength was observed between the children in the lowest and highest quartiles of maternal vitamin D status; if this difference were maintained into adulthood, given the association between grip strength and falls and fractures in later life, it might translate into an 8% reduction in falls risk and a 6% decrease in fracture risk. Confirmation in an intervention study is now required and the MAVIDOS study will be able to contribute to this knowledge gap as grip strength has been assessed in the offspring at 4 years and 6–7 years of age.

## Maternal 25(OH)D Status and Obstetric Health

As with outcomes relating to foetal development, there are numerous observational studies reporting on associations between maternal vitamin D intake or serum measurement of 25(OH)D and obstetric complications, including gestational hypertension (GHT), pre-eclampsia (PET), gestational diabetes (GDM), timing and mode of delivery. The findings of these studies are inconsistent, but there is great variation in the timing of 25(OH)D measurement, definitions used for VDD and the outcomes and covariates included [129]. In particular, consideration of the population studied and covariates used might be important to the observed relationships: it is well recognised that obese individuals have lower 25(OH)D status, and also a higher incidence of GDM, GHT, PET, caesarean section and preterm delivery [130, 131]. Similarly African American women are more likely to require delivery by Caesarean section and to have pregnancies complicated by PET and preterm labour [132]. Whether these outcomes can truly be attributed to lower 25(OH)D and therefore prevented by vitamin D supplementation must be established through intervention studies, yet currently there is a paucity of good-quality interventional data with regard to these outcomes. In particular, the majority of studies are likely to be underpowered to detect a difference in these outcomes. In the UK, GDM complicates approximately 4.5% of pregnancies [133] and therefore, to detect a 50% reduction in this incidence with 80% power at the 5% significance level, 1010 women would be needed in each study arm. As PET complicates fewer pregnancies (2–3%), even larger study numbers are needed. Recent meta-analysis of interventional studies assessing maternal obstetric health outcomes has not found an effect of vitamin D supplementation during pregnancy on reducing the incidence of PET, GDM, preterm birth, mode of delivery or risk of stillbirth [86]; however ongoing trials will be able to contribute further data on these outcomes.

## Vitamin D Supplementation in Pregnancy and Childhood

It is without doubt that both childhood rickets and neonatal hypocalcaemia secondary to VDD are preventable diseases, and this alone justifies the consideration of vitamin D supplementation in childhood and pregnancy despite the uncertainty around links between vitamin D status and other outcomes.

**Fig. 4:**
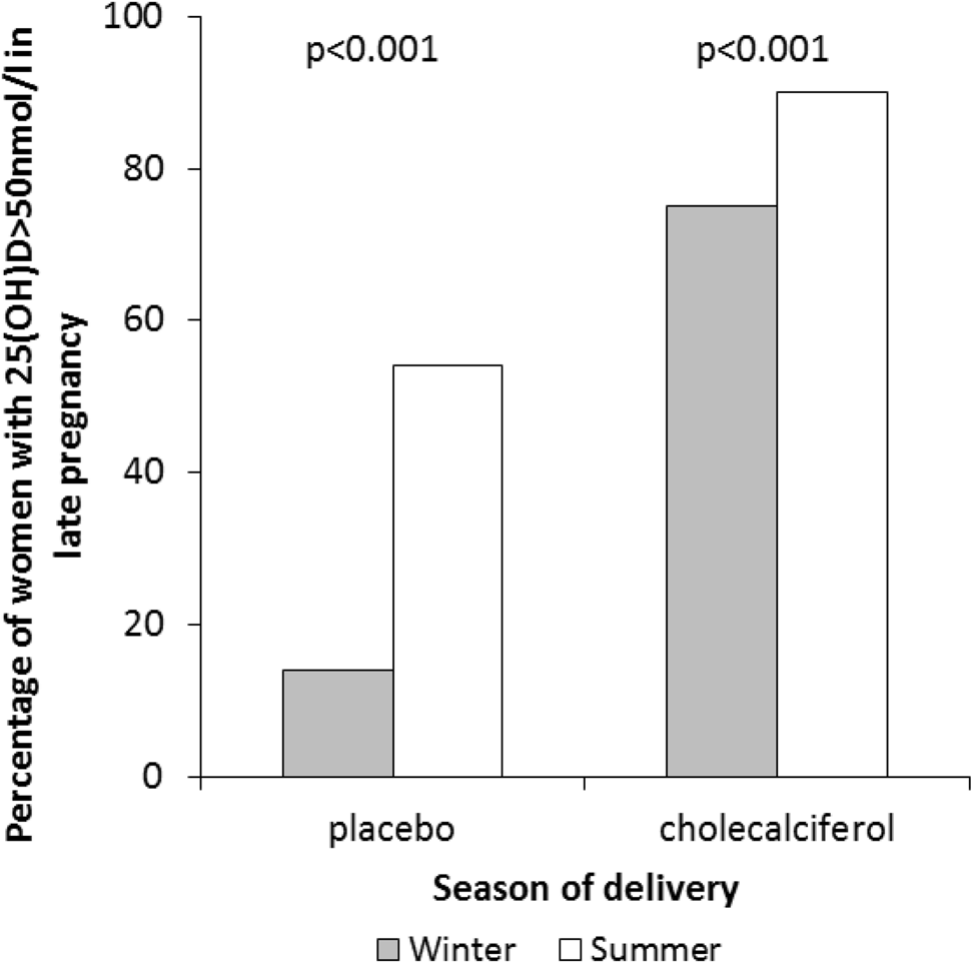
Proportion of women achieving vitamin D replete status [25(OH)D > 50 nmol/l] in late pregnancy stratified by randomisation to placebo or 1000 IU/day cholecalciferol and season of delivery. Winter was defined as December–May. Using data reported in Moon et al. [139]

A number of national and international guidelines recommend vitamin D supplementation during childhood and pregnancy, mostly between 400 and 600 IU/day [134–137], although the Canadian Paediatric Society recommend up to 2000 IU/day during winter months [138]. However, there is evidence to suggest that in pregnancy this may be insufficient to achieve vitamin D replete status in many women. In the MAVIDOS study conducted in three centres in the UK at latitudes between 50.9 and 53.4°N, 83.3% of women randomised to 1000 IU/day cholecalciferol achieved a 25(OH)D > 50 nmol/l at 34 weeks gestation, compared with 35.6% of women in the placebo group. Moreover, in both treatment groups, the proportion of women who were vitamin D replete (> 50 nmol/l) in late pregnancy was lower in those who delivered in winter (December–May) (Fig. 4), highlighting that in the UK, 1000 IU/day cholecalciferol during pregnancy does not abolish seasonal variation in 25(OH)D status [15, 139]. Furthermore, in this study, women with 25(OH)D < 25 nmol/l at baseline were excluded from participation and therefore the true repletion rate across the general population would be expected to be lower than observed in the trial population. As such, if the aim of supplementation is to increase maternal 25(OH)D to > 50 nmol/l, then it is likely that 400 IU/day will not achieve this in many women. Similarly a study of children in Denmark (55°N) suggested a total vitamin D intake of 800 IU/day would be needed to maintain a serum 25(OH)D > 50 nmol/l in late winter [140]. However, whilst many observational studies do suggest that achieving higher 25(OH)D levels might have beneficial effects, such a change in public health policy should be based on established benefits in high-quality RCTs. It is also important to be certain that in addition to benefits, that a higher supplementation dose will not be harmful. Literature with regard to falls risk in older individuals suggests that moderate doses of vitamin D (600–1000 IU/day) may have a beneficial effect whilst high bolus doses increase the risk of falls [141]. Although antenatal supplementation with cholecalciferol doses up to 4000 IU/day and childhood supplementation with 800 IU/day did not result in hypercalcaemia or clinical side effects in clinical trial settings [80, 140], until a clear benefit of higher dose antenatal and childhood supplementation has been demonstrated, such doses should not be recommended.

**Fig. 5:**
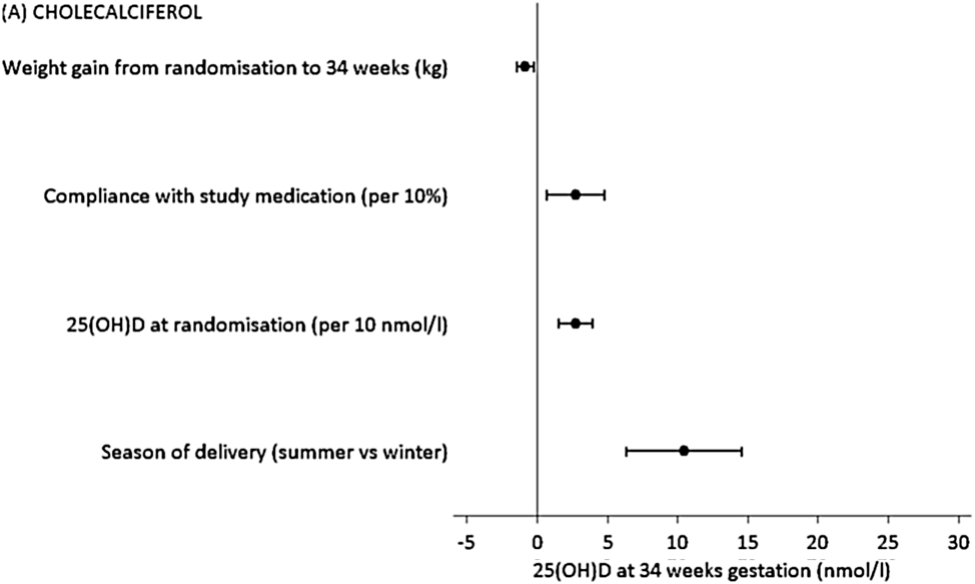
Independent determinants of maternal 25(OH)D at 34 weeks of gestation in women randomised to 1000 IU/day cholecalciferol. Shown as change in 25(OH)D per unit predictor (*β* and 95% confidence interval). From Moon et al. [139]

It is also important to recognise that the serum 25(OH)D achieved following supplementation has also been shown to reflect individual characteristics. In the MAVIDOS study, factors significantly associated with maternal 25(OH)D at 34 weeks gestation following cholecalciferol supplementation were season of delivery, baseline 25(OH)D at randomisation (14 weeks gestation), compliance with the medication and weight gain from 14 to 34 weeks [139] (Fig. 5). The latter is consistent with previous observational findings suggesting that greater weight gain in pregnancy is associated with a reduction in 25(OH)D between early and late pregnancy [15]. Ethnic differences in the response to supplementation are also apparent: for example, Hollis et al. found that even with 4000 IU/day cholecalciferol during pregnancy, women of African American ethnicity had lower 25(OH)D than Caucasian or Hispanic Women [80]. This may in part reflect genetic variation in vitamin D metabolism and indeed a number of SNPs in or near to genes in the vitamin D metabolism pathway have been identified that appear to modify the response to vitamin D supplementation [142]. In the MAVIDOS study, the SNPs found to be associated with baseline 25(OH)D in pregnant women (rs12785878 in *DHCR7*, encoding 7-dehydrocholesterol reductase in the skin), differed to those associated with the 25(OH)D achieved post supplementation (SNPs in genes encoding 25-hydroxylase and DBP) [143]. As we move towards an era of personalised medicine, consideration of individual characteristics and the presence of “at-risk” alleles may be beneficial [144] and future research studies are needed to demonstrate whether dosing schedules based on such characteristics will achieve vitamin D repletion in a greater proportion of participants and improve clinical outcomes.

Demographic differences have been observed between women who did and did not take vitamin D supplementation during pregnancy [15] and are similar to those associated with reduced likelihood of folic acid supplementation during pregnancy [145, 146]. These include younger maternal age, lower educational achievement, higher pre-pregnancy BMI and maternal smoking. Somewhat worryingly, many of these characteristics are associated with low serum 25(OH)D before supplementation, and poor supplement uptake is likely to further compound the likelihood of continued deficiency during pregnancy. In the UK, free ‘Healthy Start’ vitamins are available to pregnant women, new mothers and children in low-income families, yet uptake of this provision is low, in part due to poor understanding of their importance, but also lack of awareness of the free supplementation programme [147]. In a recent survey across European countries, adherence to vitamin D supplementation in infants was higher in countries which provided universal supplementation independent of infant mode of feeding, gave information on supplementation at discharge from neonatal units and monitored adherence to supplementation at child health surveillance visits [148]. Such approaches may therefore be needed in both childhood and pregnancy to improve the uptake of supplementation and indeed, a programme of universal multi-vitamin supplementation of all children under 5 years and pregnant and lactating women in Birmingham reduced the incidence of symptomatic VDD [149]. Health professional awareness and education regarding vitamin D might also have a positive effect on supplementation usage [150].

## Conclusion

It is without doubt that low levels of 25(OH)D are common in children and pregnant women, and this can result in VDD rickets and hypocalcaemia. In recent years, there has also been increasing interest in possible functions of vitamin D in childhood, foetal development, in particular bone mineralisation and obstetric health. However, currently the majority of evidence is observational in nature and, as yet, not supported by high-quality data from interventional trials. There are, however, a large number of ongoing studies which will increase our knowledge and understanding in the near future. Nonetheless, routine supplementation with 400 IU cholecalciferol daily during pregnancy and childhood, as suggested by many guidelines internationally, is justified. Furthermore, there is evidence to suggest that certain characteristics, including genetic factors, influence an individual’s response to supplementation, though currently these are not usually taken into account when advising on whether a higher-level supplementation is necessary for particular individuals. As we move into an era of personalised medicine, future research to establish supplementation protocols based on these characteristics is needed, but perhaps more importantly, approaches to ensuring supplementation initiation and adherence must be a priority.
